# NO_x_ Emission Trading in a European Context: Discussion of the Economic, Legal, and Cultural Aspects

**DOI:** 10.1100/tsw.2001.288

**Published:** 2001-10-25

**Authors:** Chris P.A. Dekkers

**Affiliations:** Netherlands' Ministry of Housing, Spatial Planning and the Environment, Climate Change and Industry Directorate, The Hague, The Netherlands

**Keywords:** emission, emission trading, flexible instrument, nitrogen oxide, NOX, nitrogen, national emission ceilings, NEC Directive, large combustion plants, LCP Directive, integrated pollution, prevention and control, IPPC Directive, best available techniques, ALARA principle, U.K. SO2 emission, trading, cap and trade, rate–based system, performance standard, performance standard rate, PSR regulatory culture, VROM, Ministry of Housing, Spatial Planning and the Environment, Netherlands, ACE, Automated Credit Exchange, pollution

## Abstract

Emission trading is a new instrument in environmental policy. It is an alien notion in most European countries and it is often viewed with hesitation. The paper discusses the economic, legal, and perhaps more importantly, the cultural aspects to consider when one tries to explore the prospects for trading emissions of NO_X_ and other substances in Europe. Issues to be addressed are the present legal framework in Europe in relation to the national emission ceilings on NO_X_ and other substances on the basis of relevant EU directives and UNECE protocols. The paper will discuss the extent to which the legal framework within the EU imposes constraints on the design of a national emission trading scheme, and what options are available to fit emission trading into that legislative structure. The NO_X_ emission trading programme developed in the Netherlands will be used to demonstrate the various aspects in a European context.

### INTRODUCTION

For the last 4 or 5 years, and mainly as a result of the Kyoto Protocol, emission trading has been a major topic in environmental policy discussions in Europe. However, most of these discussions have been directly related to the climate change negotiations and $CO_2$ emissions, and much less to emission trading as an instrument for achieving cost–effective reductions of other emissions, such as $SO_2$, VOC, and $NO_x$. In fact, the first discussions on flexible instruments as part of the climate change negotiations showed the wide gap of understanding between the U.S. and most European countries on the usefulness, need, and desirability of emission trading. It took most countries some years to overcome this early reluctance and even now the concept of emission trading is still not accepted everywhere in Europe as a next phase in environmental policy development.

This should not have been a surprise. Emission trading, in order to function properly, requires a well–defined legislative context. And, as legislation itself is one of the most important cultural aspects of modern society, strongly tied to the norms and values held by its people, the success or failure of emission trading very much depends on the acceptance by the main stakeholders that emissions are a "normal" or unavoidable part of industrial production. In an emission trading environment, that acceptance of "normality" implies that emissions or emission performance beyond what is legally required represents an economic value

* This article represents the views of the writer, and not necessarily the views of the Ministry of Environment.

E–mail: chris.dekkers@minvrom.nl
© 2001 with author.

that may be transferred to another facility in a similar way as other "market products" may be transferred from one company to the other. The new aspect is that so far in most societies, and thus in most environmental legislative systems, emissions and/or emission performance beyond an agreed target has not been defined as an economic transferable good or value. Indeed, until quite recently they have not even been thought of as such, by industry, politicians, or governments. And although by now the idea of $CO_2$ emissions trading has become widely accepted in most societies with environmental pressure groups participating in the international negotiations on $CO_2$ emission trading, the interest in Europe for the trading of other emissions, such as $SO_2$ and $NO_x$, is still very low.

This may change very rapidly in the next few years as soon as the discussion starts on how to implement the requirements of the recently agreed National Emission Ceilings (NEC) Directive[1], and the consequences of complying with the EU air quality requirements on ozone become apparent. To what extent is Europe ready to start emission trading and what are the possible barriers for implementing such a new approach? Two examples will be discussed. The first one involves the efforts undertaken in the U.K. to develop a system of tradable $SO_2$ permits. The project finally had to be stopped and abandoned in 1995 when it became clear that the parties in that discussion could not agree on the various elements of the trading programme. The second example involves the Netherlands and its development of $NO_x$ emission trading, which started in 1997. Although the Dutch industry itself was one of the promoters for the establishment of a $NO_x$ emission trading scheme, it took nearly 3 years of intense discussions among all parties involved before the various aspects of this $NO_x$ emission trading programme were sufficiently explored and the results could be agreed upon. This shows that the development of an emission trading programme is far from simple. It is therefore most useful to see what the problems are for setting up such a programme in a European context and what lessons can be learned from the experiences so far.

The cultural aspect of accepting that emissions or emission performance may have an economic transferable value is one thing. Existing legislation as a barrier or impediment to change is another. Both aspects need careful consideration. For instance, a recent internal draft proposal[2] of the European Commission outlines a directive to promote $CO_2$ emission trading within the European Community. Also this draft shows the potential conflict of legislative principles.

This paper discusses the design issues of the two examples on $SO_2$ and $NO_x$ emission trading, i.e., the abortive $SO_2$ trading in the U.K. and the $NO_x$ trading being developed in the Netherlands. It discusses these experiments with a view also to the recent draft for an EU directive on $CO_2$ trading and explores the chances for success of emission trading programmes in a European legislative and cultural context.

### EU ENVIRONMENTAL LAW AND ITS RELATION TO THE NATIONAL LEGISLATIONS OF THE MEMBER STATES

A most important element in any discussion on national programmes of emission trading is the interface between the Member States' legislative systems and European environmental law. The following European directives have a direct or indirect impact on national programmes of emissions trading:

1. Directive of the European Parliament and the Council on national emission ceilings for certain atmospheric pollutants, the so–called NEC Directive.
2. Directive of the European Parliament and the Council on the limitation of emissions of certain pollutants into the air from large combustion plants. This new LCP Directive, to be published shortly, replaces the LCP Directive of 1988[3].
3. Council Directive 96/61/EC of 24 September 1996 concerning integrated pollution prevention and control, the so–called IPPC Directive[4].
4. Proposal by the Commission for a Directive relating to ozone in ambient air[5].

#### National Emissions Ceilings (NEC) Directive

The objectives of the NEC Directive are to achieve substantial emission reductions in Europe for all four long–range, transboundary polluting substances, i.e., $NO_x$, $SO_2$, VOC, and $NH_3$. To that effect, the Directive contains national emission ceilings for these four substances. Member States have to take measures to achieve by 2010 emission reductions in line with the national ceilings for the four substances listed. For most northwestern European countries, this amounts to $NO_x$ reductions of 50%+ in 2010 compared to the emission levels in 1990. The emission trading programme developed by the Netherlands is geared toward compliance with the Directive requirements on $NO_x$. In a similar way, the Netherlands also intends to introduce emission trading programmes for $SO_2$ and VOC as cost–effective means to comply with the NEC Directive's requirements. The Directive recently came out of a long conciliation procedure between the European Parliament, Commission, and the Council. Agreement was reached in June 2001 and publication is expected in October 2001. It will come into force 12 months later. Member States will be required to set up programmes providing information on the measures in force and/or planned to realise the national emission ceilings.

#### Large Combustion Plants (LCP) Directive

In the same conciliation procedure as for the NEC Directive, agreement was also reached in June 2001 on the proposals for amending the former LCP Directive from 1988[4]. The requirements of this new Directive will also come into force by October 2002. Whereas the LCP Directive of 1988 required that Member States achieve overall reductions of $NO_x$ emissions from existing plants in the range of 40%, the new Directive requires that Member States ensure by national legislation that all combustion plants above 50 MWth comply with the emission limit values (ELVs) laid down in the Directive. The revised LCP Directive contains much more stringent ELVs for new installations as well as ELVs for existing combustion plants.

#### Integrated Pollution Prevention and Control (IPPC) Directive

The IPPC Directive requires, among other things, that Member States ensure by national legislation that each plant/installation be provided with a permit and be operated in accordance with the permit requirements outlined. The permit will contain ELVs that are based on an assessment by the competent authority of the emission values achievable by application of Best Available Techniques (BAT). Only under certain conditions are Member States allowed to prescribe ELVs in general binding rules. From the point of view of emission trading, the requirement of defining ELVs on the basis of BAT is a legally undesirable "constraint." It demonstrates the conflicting principles between a target–based approach of emission trading and the concept of enforcing BAT through the permit procedure. This will be discussed in detail in the following sections of this paper.

#### Commission's Proposal for a Directive Relating to Ozone in Ambient Air

A fourth Directive, presently undergoing a conciliation procedure between the European institutions, relates to ozone in ambient air. It aims to ensure effective protection against harmful effects on human health from the exposure to ozone and sets long–term objectives to reduce as much as possible the adverse affects on vegetation, ecosystems, and the environment as a whole. In the Netherlands, in most cases, traffic emissions are the cause of exceeding locally the $NO_x$ quality requirements of the Directive. Only in very specific situations are industrial sources expected to contribute significantly to exceeding local air quality limit values.

#### Relationship with National Legislation

All four Directives require that Member States take appropriate measures to ensure that the objectives laid down in each are met within the time scale agreed. In general Member States are required to draw up national programmes demonstrating that national legislation has been enacted to enforce the measures required by the Directives. Member States have a certain degree of freedom and some room for manoeuvre in the way these requirements are met and the designation of the "competent authority" in the sense of the relevant Directive. In some countries national legislation delegates the requirements to local or regional authorities, whereas in other countries a government agency is designated as "competent authority." In the Netherlands the national legislation delegates the requirements for permitting and enforcement to the provinces and municipalities. Provinces are the "competent authority" for permitting and enforcement of the IPPC requirements for the larger industrial facilities.

### THE U.K. EXPERIMENT ON $SO_2$ EMISSION TRADING

In a study published in 1999[7], Steve Sorrell and Jim Skea of the University of Sussex, Brighton, assessed the reasons why efforts in the U.K. to develop a system of $SO_2$ emission trading in 1994 to 1995 failed. At the end of their analysis, the authors compare the reasons for success of the Acid Rain Programme in the U.S. with the reasons why the efforts in the U.K. had a negative result. They list a number of determinants of success or failure that are most relevant in the European discussion on emission trading. Their main conclusion is that the failure to develop $SO_2$ emission trading in the U.K. was the result of a number of "conflicts":

1. Conflict of regulatory principles
2. Conflict of regulatory culture
3. Conflict over system and the determination of emission quota

In the end these conflicts resulted in a situation of regulatory uncertainty in the U.K., which was intensified by the lack of adequate political support. In this paper the analysis developed by Sorrell and Skea is followed to compare the results of their analysis for the U.K. with the situation in the Netherlands and to draw some general lessons from it.

#### Regulatory Principles

Sorrell and Skea summarised their analysis in a table, comparing the differences in regulatory principles in the U.S. and the U.K. Table 1 below, which has been somewhat adjusted for the purpose of this paper, highlights the differences in regulatory approaches that play an important role in the discussion on emission trading. It focuses the attention on the very basic differences between the target–based approach of emission trading vs. the technology–driven, "command and control" approach that is still generally practiced in Europe and enhanced by the IPPC Directive.

The table provides only an "image" of the basic differences between the two legislative systems and enforcement policies. The analysis of Sorrell and Skea shows that also other elements, i.e., regulatory culture and design issues, are of crucial importance.

#### Regulatory Culture

As to the differences in regulatory culture between the U.S. and the U.K., Sorrell and Skea make the observation that in the U.S., emission trading was developed as an alternative to a rigid and complex regulatory system of "command and control" of predominantly uniform standards. In the U.K., the regulatory system would better be described as predominantly "flexible and informal," with a preference for individually negotiated, site–specific standards. In the U.S., the regulatory culture was characterized by suspicion of industry self–regulation and by extensive use of litigation and minimum administrative discretion. In the U.K., industry self–regulation is encouraged, court action is seldom taken, and a maximum of administrative discretion is used. In line with this "arche–typing" of differences between the U.S. and the U.K. regulatory culture, relationships between industry and the regulator in the U.S. is characterised by confrontation, whereas in the U.K. this relationship is one of cooperation. In the U.S. there is freedom of information, whereas confidentiality of information is the main line of the regulator in the U.K.

**TABLE 1**
**Comparison between Target–Based Emission Trading and the Traditional "Command and Control" Technology–Driven Approach as Prescribed by IPPC**

| Target Oriented: Emission Trading | Technology Oriented: Integrated Pollution Control |
|---|---|
| **U.S.**: | **Europe:** |
| Offset policy first introduced in 1976 | Use of technology–based principles |
| **Economics based:** | **Engineering based:** |
| Pollution arises from an absence of well–defined property rights; command and control is becoming more and more costly and ineffective | Abating emissions is a technological problem, a matter for engineers and scientists; emission reductions through enforcement of new technology |
| **Target based:** | **Technology based:** |
| Overall pollution target with no specification of individual technologies or standards | Minimisation and maximum abatement of emissions/pollution through application of BAT |
| **Hands off:** | **Hands on:** |
| Technology decisions are the responsibility of the individual firms | Regulator is involved in the technology decisions through permit procedures |
| **Wide system boundary:** | **Narrow system boundary:** |
| Aggregate target can be as wide as a sector, industry, or geographic area | BAT applies to the individual process and/or installation |
| **Single pollutant/medium:** | **Multipollutant/media:** |
| Controls a single polluting substance in a single medium | Controls releases of a wide range of substances to all three media—air, water, and land—in an integrated manner |
| **Flexibility via the market:** | **Flexibility through negotiations:** |
| Installation operators can seek flexibility and reduced costs through trading in the permit market | Installation operators can seek flexibility and reduced costs through negotiations with the permitting authority |

The description above is admittedly a cursory and purposely broad depiction of what is the normal situation in the U.S. and the U.K. Moreover, since the first analysis along the above lines was carried out in 1986, much has changed as a result of the debate on eliminating the most rigid elements of the U.S. regulatory system, whereas in the U.K. the regulatory style has changed also as a result of the requirements of the European legislation.

### Conflict over System and the Determination of Emission Quota

The allocation of emission quota and the selection of the concept of emission trading is generally regarded as one of the most thorny issues of any emission trading programme. Sorrell and Skea conclude that the issue of quota allocation and the question of what would be a just and equal burden upon the sectors played a central role in the failure of the $SO_2$ programme in the U.K. Although the details of the various modes of $SO_2$ allocations are most relevant for the parties directly involved in the negotiating process, i.e., the ministry of environment, HM Inspectorate, and industry, from an analytical point of view it is relevant to see that the design issues, the guiding principles, and the negotiating process become intractably confusing if there is not a clear picture of the most critical issues at the outset. From the analysis presented by Sorrell and Skea, it appears that the direction of the policy discussion shifted during the process, and that the allocation issues and various alternatives became intractably intertwined. Halfway through the process major differences of opinion between parties emerged on how the system should be designed and be made compatible with policy objectives and with national and international legal constraints. Moreover, the perception of the seriousness of the $SO_2$ problem and therefore also of the urgency of emission trading shifted during the negotiating process as a result of the 'Dash for Gas', i.e., the major shift from coal towards gas firing in the U.K. power industry during 1992 to 1996. As a result, $SO_2$ emissions decreased much faster than had been anticipated at the start of the policy discussion.

### Compatibility with IPPC

The approach selected in the U.K. at the start of the negotiating process also seems to have been developed with too little attention to the questions of compatibility and the limitations of existing national and European legislation. For instance, grandfathering emissions on the basis of historic emissions is probably most difficult to bring in line with the concept of BAT and other principles of environmental policy enshrined in European environmental legislation. It appears that halfway through the debate on $SO_2$ emission trading, the U.K. Inspectorate proposed that quota should be redistributed on the basis of historical fuel use, regardless of fuel type. Sorrell and Skea consider the proposed approach as simple and consistent, justified under the polluter pays principle and rewarding 'clean plants' over more polluting plants. The one fuel concept has some resemblance to the system proposed for the Netherlands. Probably such an approach would have been easier to bring in line with the principles of applying BAT. However, these proposals were introduced too late in the negotiating process, at a moment when positions had already hardened. Anyway, the parties involved did not accept the proposals. Sorrell and Skea suggest that the outcome of the debate would have been different, if only the Inspectorate had introduced its proposals earlier in the debate.

### $NO_x$ EMISSION TRADING IN THE NETHERLANDS

In a discussion of the $NO_x$ emission trading programme developed by the Netherlands, it is useful first to assess where the Netherlands would fit into the analysis of Sorrell and Skea and their comparison of the regulatory principles and culture.

#### Regulatory Principles

The situation in the Netherlands differs to some extent from that in the U.K. Discussions on flexible approaches started in the Netherlands in the early 1980s, not too long after the first experiences in the U.S. Moreover, the "bubble" concept for an industrial facility or site, whereby all emission sources from an industrial facility are considered as being one source, is well accepted in the Netherlands. There is quite some experience with flexible approaches in covenants and similar agreements with industry, and economic and technical arguments have been integrated in Dutch legislation and administrative procedures. Environmental planning started early in the 1980s and environmental targets have played a major role in policy development by the Ministry of Environment. This target orientation is well enshrined in the National Environmental Policy Plans. However, realisation of the targets is mainly delegated to the regional authorities (provinces) for which the permit is the main instrument to realise emission reductions. Examples of "Hands Off" as well as of "Hands On" approaches can be recognised in practical situations. As to the wide or narrow boundaries, there is in general a positive attitude to relate environmental solutions to the appropriate aggregate level. Integration at the level of the permit is standard practice. For certain problems, like the emissions of $NO_x$ and other emissions causing transboundary pollution, it is a well–established practice in the Netherlands that environmental targets at a national level are formally prioritised by law and therefore obligatory and overruling other environmental issues at the level of the permitting authority. There is no experience with emission trading. For the various industrial sectors, long–term emission reduction targets have been set, which are then used as a "guide" for the environmental agenda of the individual companies in that sector. In fact this is part of the regulatory culture.

The conclusion is that conflict of regulatory principles that apparently played such an important role in the U.K. went by largely unnoticed in the Netherlands. The notion in the U.K. that emission trading is severely constrained by the IPPC Directive was not realised until quite recently by the various partners in the Netherlands' discussion on emission trading. The question of how to integrate the major design elements of the $NO_x$ emission trading programme into the specific permitting requirements of the IPPC Directive have raised some major issues which will be explained in sections further on in this article.

#### Comparison with the Regulatory Culture in the Netherlands

Simple comparisons are illustrative of larger themes. A crude assessment of the regulatory culture in the Netherlands along the same lines of Sorell and Skea's assessment shows that since the early 1970s, the Netherlands developed a very extensive system of legislative requirements, uniform procedures, and standards in advance of and sometimes in parallel with European legislation. Nevertheless, throughout the 3 decades, the legislative requirements also maintained a certain discretionary flexibility on the side of the regional or local authority, respecting thereby the traditional and delicate "balance of power" between the central and regional authorities, which is very typical for the Netherlands' system of public governance. Furthermore, self–regulation is a well–accepted and historically determined element in the regulatory culture in the Netherlands. Court action against an industrial enterprise is not often used: discretion is the "accepted" rule. As in the U.K., relationships between the regulator and the industry are characterised basically by cooperation. Different from "normal" practice in Europe, environmental information is freely available with free access to the individual permits and their requirements. All industrial facilities above a certain threshold size or production capacity are by law obliged to publish a yearly environmental report.

The situation in the Netherlands can be described as somehow "covering middle ground" between the U.K. and the U.S.: uniform standards combined with discretionary flexibility by the permitting authority, public accountability, and free access to information combined with self–regulation and discretion in solving problems when they arrive.

#### Design Issues of the Dutch Trading Programme on the $NO_x$ Emissions of Industrial Facilities

At the early start of the discussions, in 1997, a choice was made on the very basic elements of the $NO_x$ emission trading programme. There was a clear view among policy experts in the Ministry of Environment that emission trading on the basis of cap and trade, i.e., grandfathering on the basis of historic emissions, would not be compatible with the national Environmental Management Act (EMA) or the European IPPC Directive. The EMA requires that permitting authorities apply the ALARA principle (*A*s *L*ow *A*s *R*easonably *A*chievable), while the IPPC Directive requires i.a. that industrial plants (installations) apply BAT. Both principles aim at achieving a high level of protection, recognising that an evaluation by the competent authority of the technical solutions to reduce emissions also involves an economic judgment by the competent authority of the reasonability of the cost implications. The discussions in 1997 centred on the question of how to design an emission trading programme aimed at achieving the long–term emission targets and geared towards realisation of target–related reductions by the facility as the "decisive" unit. The first observation was that 80% of the $NO_x$ emissions are directly related to the use of fossil fuels in industrial facilities. This recognition led to the concept of "credit trading," whereby facilities that would perform better than the annual targeted "average" or "base line emission level," later on defined as a "performance standard rate," would be allowed to trade their surplus "reduction" with facilities that are unable to reduce their emissions at reasonable cost to that baseline or performance standard rate (PSR). Although the first concepts of emission trading started with defining two "baselines," one for gas–fired installations and one for other fuels, during subsequent discussions with industry this "two fuel concept" was discarded as not effective in an emission trading environment. It was argued that making a distinction in the PSR between gas and coal/oil would unduly penalise companies wanting to reduce their emissions by shifting to gas or another clean fuel. Basically, this "one fuel" concept and an annual declining PSR (until 2010), equal for all facilities, has been the main conceptual line in the programme. All parties finally accepted this line in December 2000, when the other elements of the emission trading programme were also agreed upon between the Ministry of Environment, the provinces, and the various sectors of industry. In February 2001 Parliament[8] was informed, and in May 2001 its Committee on the Environment endorsed the main lines of the scheme. The various technical aspects of the Dutch programme have been presented at the N2001 conference by Mr. Bill van Amburg of Automated Credit Exchange (ACE) of California, who has been retained as consultant in the development of the Dutch Emission Trading Programme. Furthermore, the Programme has been well documented in various reports, which are freely available in hard copy or electronic format[9].

### Aspects of National Law

A major question in the early discussions with industry was whether and how emission trading could be fitted into the existing EMA. Several options were reviewed. At first it was thought that emission trading should be based on a standard requirement applying to all facilities, and that facilities seeking to participate in "cost sharing" would enter into a covenant, with a single emission requirement applying jointly to all participating facilities. However, the idea of a covenant was subsequently abandoned as impractical and not legally sound. Another consideration was that in the end, industry preferred a legally secured system of emission trading.

The system, as it has been further refined, centres around the uniform PSR defined as a statutory standard established by order in council and applying to all larger facilities. A facility can comply with the PSR either by taking measures itself to reduce its emissions as required, by purchasing $NO_x$ credits, or by a combination of these two options. A facility with combustion plants falling under the LCP Directive (≥50 MWth) cannot, however, meet its environmental obligations exclusively by purchasing $NO_x$ credits. It will have to comply with the LCP–emission requirements on a per installation basis through new or existing physical measures in–house.

During the further development of the system, the major legal question then became whether a system of allocating emission allowances to facilities and allowing them to sell unused allowances or buy allowances from other facilities where their own are exceeded would be compatible with the EMA. More specifically, whether emission trading would not violate the ALARA principle enshrined in the law. Asked to resolve this question, the Council of State clarified that a system of emission trading could be helpful in fulfilling the international obligations, but also concluded that the proposed system of $NO_x$ emission trading could not be implemented by means of an order in council under the present law. The Council took the view that there is a fundamental incompatibility between the philosophy of the EMA and the concept of tradable rights. The Council advised that separate legislation would be necessary. Furthermore, the Council found that the EMA is based on principles that are diametrically opposed to the intrinsic characteristics of an effective system of emission trading. It took the view that the EMA is not directly aimed at reducing emissions at a national level, and also that the concept of transferable pollution is quite alien to the facility–oriented approach of the EMA. In light of the Council's information, the Minister of the Environment decided that in November 2001 the EMA would be amended so as to enable emission trading (including $NO_x$) in the future.

### $NO_x$ Emission Trading Aimed Compliance with the NEC Directive

As explained before, the European framework of directives is of major importance with respect to national programmes on emission trading and its intended policy objectives. Two directives are of crucial importance.

The recently agreed National Emission Ceilings (NEC) directive requires that in 2010 Member States have reduced their national emissions of $SO_2$, $NO_x$ VOC, and $NH_3$ to the ceilings agreed in the new directive. The national ceiling of $NO_x$ emissions for Netherlands amounts to 260 kilotonnes $NO_x$ in 2010, of which the government has allocated 55 kilotonnes to the industrial facilities intended to participate in emission trading. Table 2 shows the contributions in the draft national reduction plan required from the sectors, in perspective of their emissions in 1995. It shows that all sectors have been charged with similar reduction targets of approximately 55% in 2010 compared to 1995 emission levels. A contingency has been built-in as an additional assurance that in 2010 the national obligations with respect to $NO_x$ are being fulfilled.

The second directive of equally crucial importance is the Integrated Pollution Prevention and Control Directive (IPPC, 96/61/EC). It provides the basis for the permit procedure with emphasis on applying Best Available Techniques (BAT), which so far has been regarded by the European Commission as the main instrument by which Member States should ensure the required emission reductions from industrial activities. It is assumed that prescribing in the permit of industrial facilities emission limit values on the basis of the application best available techniques will ensure the emission reductions possible and achievable in each individual situation. In fact this assumption is build on a false perception of the real world of permitting, as will be explained further on.

## EMISSION TRADING WITHIN THE EUROPEAN LEGISLATIVE FRAMEWORK

From early on, European policy on the reduction of air pollution has been developed along two main lines of thought, i.e., the target–oriented approach vs. the technology–oriented approach. In the 1988 LCP Directive (88/609/EEC), the technology–ori-

**TABLE 2**
**The Netherlands' National Emission Ceiling for $NO_x$ as Imposed by the NEC Directive, and the Reduction Contributions from Industry in Relation to the Other Sectors**

| $NO_x$ Emissions (in ktonnes) | Targets 2010 | Emissions 1995 |
|---|---|---|
| **Larger industrial facilities in emission trading system** | **55** | **120** |
| Smaller industrial facilities | 10 | 20 |
| **Total industry** | **65** | **140** |
| Traffic and other sources | 166 | 350 |
| **Total emissions** | **231** | **490** |
| Contingency | 29 | |
| **National emission ceiling [NEC directive]** | **260** | |

ented approach, led to uniform ELVs for new combustion plants ≥50 MWth, detailed per type of fuel and size of the installation. However, for existing combustion plants, the target–oriented approach was adopted, specifying per Member State the 1993, 1998, and 2003 reduction targets and emission ceilings. In a similar dual approach, the recently agreed NEC Directive dictates national emission targets to be reached in 2010, while the newly amended LCP Directive is fully technology–oriented by defining new, more stringent ELVs for new plants as well as emission limits values for existing plants that were built before 1988. And while the LCP Directive aims at defining uniform emission standards as minimum standards over the whole EU, somehow related to the emission reduction targets at a national level, the IPPC Directive (96/61/EC) is directed toward and fully supportive of the technology approach. It requires that the competent authority ensures that the installations covered by the IPPC Directive are subject to specific permit requirements and ELVs based on applying BAT. However, it is not always clear whether the "installation" in the sense of the IPPC Directive aims at the facility or the individual combustion plant or the individual emission source. This ambiguity in the definitions and the logic assumed behind the various articles of the directive adds to the confusion. However, the IPPC Directive leaves the competent authority little room for manoeuvre or flexibility in formulating the requirements to be imposed on the facility. Article 9, sub 4 is crucial: "*. . . the emission limit values and equivalent parameters and technical measures* . . . < in the permit> . . . *shall be based on the best available techniques . . . taking into account the technical characteristics of the installations concerned, its geographical location and the local environmental conditions. In all circumstances, the conditions of the permit shall contain provisions on the minimization of long distance or transboundary pollution and ensure a high level of protection for the environment as a whole.*"

The last sentence is most relevant with respect to $NO_x$ emission trading. In fact, the competent authority is required to assess what is technically feasible at the installation, and to formulate ELVs based on that assessment. The major question that the legislative experts of the Ministry of Environment are faced with is how to structure $NO_x$ emission trading around the concept of a yearly declining, uniform PSR per facility, while respecting the strict requirements of IPPC and BAT at the level of the "installation." The Netherlands have proposed to draft a new Order in Council under Dutch law that will contain the type and fuel specific $NO_x$ requirements of the recently agreed amendment of the 1988 LCP Directive as minimum requirements per individual combustion installation. In a similar way, and analogous to combustion plants, minimum requirements for a total of 15 specific processes from the chemical and the steel manufacturing industry will be formulated in this Order of Council. Thereto, use will be made of Article 9, sub 8 of the IPPC Directive that states: "*Without prejudice to the obligation to implement a permit procedure pursuant to this Directive, Member States may prescribe certain requirements for certain categories of installations in general binding rules instead of including them in individual permit conditions, provided that an integrated approach and an equivalent high level of environmental protection as a whole are ensured.*"

The approach of defining minimum ELVs in the Order in Council would ensure that the provisions of the LCP and IPPC Directives would be implemented without compromising the concept of emission allowances. However, it is not certain whether this approach suggested by the Netherlands fits with the "concept" of the IPPC Directive.

### Environmental Benefits of the IPPC and BAT Approach vs. Emission Trading

A major question in the discussion with the Commission is also what the material environmental difference is between the two approaches, i.e., the reductions resulting from the requirements in the LCP and IPPC Directives compared to the reductions achieved by the intended PSRs in the Dutch system of emission trading. In theory there should be no difference. From a purely economic point of view, reductions resulting from applying the permit procedure on the basis of the ALARA principle in the Netherlands' EMA or the principle of BAT on the basis of the IPPC Directive should be similar to the reductions achieved by emission trading on the basis of PSRs.

Both the ALARA principle and the BAT principle aim to achieve a high level of environmental protection. Both principles recognise also that an evaluation of the possibilities to reduce the emissions as low as possible involves a technical judgment at the individual process unit, plant, or emission source of what is technically feasible as well as an economic assessment of the costs involved. However, IPPC starts from the premise that the competent authority has sufficient if not full information to make that "perfect" judgment on technical feasibility and economic reason. The fundamental question is, however, especially on such issues as the techniques to reduce or to abate $NO_x$ emissions in a complex facility, whether in most practical situations the competent authority has or has access to the technical knowledge and/ or information to make that judgment. In the IPPC Directive a provision is made to address the issues of information on abatement techniques. Under article 16 of the Directive a forum[10] for the exchange of technical knowledge has been established. This is however only a partial solution. There remains always a fundamental gap of information between the operator of the facility and the competent authority.

Emission trading addresses that "information gap." With emission trading on the basis of performance standards, it is the operator of a facility who makes his own assessment of what is technically feasible in his situation, and what is economically acceptable and/or reasonably achievable against the financial pressure that is put on him by the market price of the credits exchanged. That financial pressure is the same for all emitters, and all facilities are in the same position to make similar assessments. Moreover, in a system of yearly declining performance standards, the operator will make that assessment every year anew to see whether further emission reductions are technically and economically feasible, thereby seeking continuous $NO_x$ improvements as aimed by the IPPC Directive. In that sense the PSR in the Netherlands' system of emission can also be seen as the BAT–related ELV at the level of the facility, be it with a built–in flexibility to account for technical and economic differences around the average cost for the industry to achieve the emission reduction target.

### Incorporating Emission Trading in European Environmental Legislation and Instruments

Four observations can be made with respect to incorporating emission trading in this European legislative framework.

1. A first observation is that the basic structure of the European legislative framework on air pollution was laid down years ago, at a time when emission trading was discussed only in scientific circles. Until quite recently emission trading was not considered a realistic option in European environ-mental policy development. The whole idea to incorporate in the permit ELVs for substances with primarily long–range and transboundary effects could be regarded as outdated already at the moment of the drafting of the IPPC Directive. In hindsight, it may well have been a conceptual error. One could well argue that national emission ceilings require a direct legislative translation into PSR imposed on the polluting activity or facility, instead of connecting it to technology–derived emission levels, as implied by the IPPC. Experience in the Netherlands, probably not different from that in other Member States, has shown that in the past the competent authorities, charged with the permitting procedures on the larger facilities covered by the IPPC Directive, have been unable to enforce the national $NO_x$ emission targets by means of requesting "best available technology" in the permit. The basic problem is in fact that only in the case of a permit request by the owner of the facility, the competent authority is in a bargaining position strong enough to require major adaptations on an existing combustion plant or process unit. Moreover, various studies have indicated that also in the far future, 90% of all emission reductions will have to come from existing plants, built some 30 to 40 years ago. The cost effectiveness of measures at existing plants vary greatly, and there is no "objective" instrument to assess where and when $NO_x$ emissions at such a large number of installations can be reasonably requested by the competent authority and realised in the permit to such low levels as to come close to the emission targets of the NEC Directive. In that sense "command and control" is ultimately doomed to fail. Perhaps a more philosophical observation is that IPPC, with its strong emphasis on applying BAT everywhere in the EU, seems more concerned with a technology–oriented "level playing field" in industry than with achieving emission reductions and environmental targets where these are needed. Once a certain basic level of environmental performance has been reached, the ambition to impose BAT by a command and control type of permitting will in the end and unavoidably lead to an arbitrary treatment in the practice of permitting.
2. Also in the case of other emissions, most notably those of $CO_2$ and other climate change emissions, there is an urgent need for a more flexible approach that can be fitted in and allowed or facilitated by the IPPC and its various requirements. Benchmarking for energy efficiency is just an example of a more flexible approach to achieve environmental targets. Recently, an unofficial draft proposal for a European $CO_2$ emission trading system has been circulated among industry and policy experts. From this draft proposal it is obvious that the European Commission also struggles with combining emission trading with the requirement of BAT equivalent emission values in the IPPC permit. The idea is that each installation as defined in the IPPC Directive is to receive a permit to emit greenhouse gases, and that on the basis of the permit the national government allocates to each facility/installation the emissions of $CO_2$ for a period of 3 (2005 to 2007) and then 5 years (2008 to 2012). The allocation will be based on a national allocation plan requiring notification to the Commission. Interestingly, however, the proposal contains a draft article as an amendment to the present IPPC Directive, stating that following sub–paragraph should be added to paragraph 3 of Article 9 of Directive 96/61/EC: *"the permit shall not include emission limit values for direct emission of carbon dioxide from activities that are included in Annex I to Directive xx/ xxx/EC establishing a framework for greenhouse gas emission trading in the European Community"*. This proposed article clearly demonstrates that the Commission has recognised that the IPPC Directive does not provide for the flexibility required for an emission trading system to function properly. Moreover, the administrative procedures and model of decision making required by the IPPC Directive are not formulated in such a way as to allow for a kind of guidance and governance "at a distance" whereby the owner/operator decides for himself what is technically feasible in his facility, even though the final environmental result would be better than the case whereby the competent authority quasi–decides on the reductions through ELVs in the permit.

3. Moreover, the European Commission's programme "Clean Air For Europe (CAFÉ)"[11] formally launched in June 2001 aims at establishing a coherent, long–term, thematic strategy and integrated policy to combat air pollution. The Commissions' intentions are "*to monitor the implementation of, evaluate the effectiveness of and, by the latest in 2004, review the existing legislation, taking account of particular problems in implementation identified by Member States.*" The European Council stressed "*the importance of the review of the air quality directives and the directive on national emission ceilings as elements of a more complete and integrated result–oriented air quality strategy with the aim of not exceeding identified critical levels and loads*". In view of the well–accepted recognition that emission trading is a promising, new, and cost–effective instrument in the abatement of certain air pollutants, and because the IPPC Directive is not fully supportive of emission trading, the IPPC Directive should also be scrutinised and reviewed within the CAFÉ programme on its environmental effectiveness together with and in conjunction with the other directives.
4. Within that same context it would be most useful to consider how the relationships between the national environmental targets and air quality objectives should be further developed and what role and responsibilities should be assigned to the various parties involved, i.e., national governments, permitting authorities, industry, and nongovernmental organizations such as the Dutch environmental group Nature & Environment. Emission trading should be seen as one of the more promising developments in the framework of industrial environmental management and as a conceptual, sound approach towards self–regulation. Moreover, it should be regarded as part of a modern and mature environmental policy framework in which companies strive for continuous improvement and control of environmental performance in environmental management systems like ISO 14001 and EMAS. In such a policy framework, the environmental authorities are able to focus on performance instead of technology. Licensing procedures in such a system may become less detailed and more focussed on improving and maintaining environmental quality. Such a system fits well into a modern society with corporate citizens that aim for sustainable development and balance the interests of people, the planet, and their own profits toward long–term survival of all three.

### CONCLUSIONS

Getting agreement on an emission trading scheme requires a number of very difficult changes in people's perceptions and attitudes, and sometimes even a complete reversal of very firm convictions of how problems should be solved. It requires industry's acceptance of the ambitious emission targets for future operations, of the costs involved, and of the estimates and assumptions used for assessing these costs. Industry must learn that the trading of credits is an additional and most useful instrument for compliance, and an alternative to investment in physical measures. It also requires acceptance of the need to redefine the responsibilities of the various parties and interest groups in a system of emission trading. Industry must realise that emission trading requires a more proactive environmental attitude, whereby it will be held responsible for achieving the targets set in the programme and strict financial penalties will be imposed for noncompliance. In a system of emission trading there is no room for negotiating special deals and no room for excuses or unforeseen delays. Achieving the required reductions and taking environmental measures becomes a prime responsibility of the industry itself and each step industry takes will be disciplined by market forces. But in addition the "traditional" role of the competent authorities will be affected by emission trading. They will no longer guide or force industry toward taking abatement measures. Not everybody will take such a drastic change in attitudes and responsibility for granted. In short, emission trading involves much more than most people realise at first.

A major question, then, is whether in most European countries the environmental awareness of the industry and the willingness of the competent authorities have progressed far enough to allow for the next step in environmental management and policy development. Another question is whether the various parties have enough stamina to make emission trading work. To achieve agreement, parties must have a clear concept at the start of what the basic elements of the emission trading system should be and what set of principles should be used to guide the allocation, as well as a straightforward strategy to reach consensus or acceptance between the parties affected. It should be well understood that a first successful requirement for emission trading is broad acceptance of the fact that there is a problem that cannot be solved by the traditional command and control approach.

The Dutch experience differed from that in the U.K. in that the need for an emission trading programme for $NO_x$ became well accepted by most, if not all, interest groups during the course of the negotiating process. The urgency of emission trading and justification for the stringent emissions targets in the programme were enhanced by the international negotiations on national emission ceilings in the framework of the UNECE Convention on Long–Range Transboundary Air Pollution and the recent agreement on the NEC Directive. Furthermore, the experience with $NO_x$ emission trading was perceived as a most useful pilot project for changing the perceptions of the industry on how the $CO_2$ emission trading should be tackled. It led to a much more positive attitude from the part of the industry on $CO_2$ emission ceilings than was thought possible before.

Finally, emission trading involves two elements: "emissions" and "trading." In fact, the whole concept of trading aims at making certain that the emission reductions needed from an environmental perspective are realised in time and in a most cost–effective way. The trading is subordinate, but nevertheless most important. Moreover, there is a very basic economic rule which says that there will only be trade of goods if goods have a price, so there must be scarcity. The logic is then that the trading of emissions can only succeed if there is a sufficient economic incentive toward emissions to be traded, implying that emissions are no longer handed out for free. That may be the most difficult part for all parties to accept.

### ENDNOTES

1. Proposal for a Directive of the Parliament and the Council on national emission ceilings for certain atmospheric pollutants. (2000) OJ No. C56E, 34.
2. Internal draft for a Commission proposal for a directive establishing a framework for greenhouse gas emissions trading within the European Community.
3. Proposal for a Council Directive amending Directive 88/609/EEC. (1998) OJ No. C300, 1.
4. Council Directive 88/609/EEC of 24 November 1988 on the limitation of emissions of certain pollutants into the air from large combustion plants. (1988) OJ No. L/336, 1.
5. Council Directive 96/61/EC of 24 September 1996 concerning integrated pollution prevention and control. (1996) OJ No. L/257, 26.
6. Proposal for a Directive relating to ozone in ambient air. (2000) OJ No. C56E, 41.
7. Sorrell, S. and Skea, J., Eds. (1998) *Pollution for Sale, Emissions Trading and Joint Implementation*. Edward Elgar, Cheltenham, U.K.
8. Letter to Parliament of 19 February 2001 (Kamerstukken II 2000–2001, 26 578, nr. 3).
9. Studies carried out include following:
   - Choosing to Gain: "a study of the potential cost advantages of a system of tradable permits", issued by the Inter Provincial Council (IPO) under IPO Publication 105, The Hague, May 1997.
   - The Exchange Model, "A high–potential cost sharing option as an instrument to combat $NO_x$ emissions from stationary sources in the Netherlands", issued by KPMG Environmental Consulting, De Meern, The Netherlands under guidance of the Chemical Industry Cost Sharing Consultative Group, published January 1999.
   - A system of $NO_x$ emissions trading for stationary sources of the industry, issued by KPMG Environmental Consulting, De Meern, The Netherlands under guidance of the Working Group on Emission Trading, published November 2000.
   - Potential for reduction of $NO_x$ emissions at the industry, refineries and the Power Industry, and the Costs involved, by Stork Comprimo Protech, 26 October 1998. Sensitivity analysis of the cost of $NO_x$ reductions in the Industry, Refineries and the Power Industry, also by Stork Comprimo Protech, 28 May 1999. Both reports are available only in Dutch.
   - Phase I Final Report on the Netherlands $NO_x$ Trading Program, submitted to the Ministry of VROM, July 20, 2000 by Automated Credit Exchange (ACE), Pasadena, California.
   - Phase II Netherlands $NO_x$ Trading Program, submitted January 2001.
   - Phase III report on the $NO_x$ trading simulation exercise, May 2001.
10. European IPPC Bureau, Institute for Prospective Technological Studies (Seville).
11. Commission Communication COM. (2001) 245 def.

| | | |
|---|---|---|
| Received: | July | 20, 2001 |
| Revised: | October | 3, 2001 |
| Accepted: | October | 9, 2001 |
| Published: | October | 25, 2001 |
